# Dynamic of resistance alleles of two major insecticide targets in *Anopheles gambiae* (*s.l.*) populations from Benin, West Africa

**DOI:** 10.1186/s13071-020-4006-6

**Published:** 2020-03-14

**Authors:** Benoît S. Assogba, Nicole Pasteur, Patrick Makoundou, Sandra Unal, Lamine Baba-Moussa, Pierrick Labbé, Mylène Weill

**Affiliations:** 1 Institut des Sciences de l’Evolution de Montpellier (ISEM), UMR CNRS-IRD-EPHE-Université de Montpellier, Place Eugène Bataillon, 34095 Montpellier, France; 2 Faculté des Sciences et Techniques, Laboratoire de Biologie et de Typage Moléculaire en Microbiologie, Université d’Abomey Calavi, 05 BP 1604, Cotonou, Benin; 3 Institut Régional de Santé Publique, Université d’Abomey Calavi, 01 BP 918, Cotonou, Benin; 4 Disease Control and Elimination Department, Medical Research Council, Unit The Gambia at London School of Hygiene and Tropical Medicine, Fajara, Gambia

**Keywords:** *Anopheles gambiae*, Malaria, Insecticide resistance, Evolution

## Abstract

**Background:**

Insecticide resistance is a growing concern for malaria control and vector control effectiveness relies on assessing it distribution and understanding its evolution.

**Methods:**

We assessed resistance levels and the frequencies of two major target-site mutations, L1014F-VGSC and G119S-*ace-1*, conferring resistance to pyrethroids (PYRs) and carbamates/organophosphates (CXs/OPs) insecticides. These data were compared to those acquired between 2006 and 2010 to follow resistance evolutionary trends over ten years.

**Results:**

We report the results of a 3-year survey (2013–2015) of insecticide resistance in 13 localities across the whole country of Benin. Permethrin (PYR) resistance was found in all populations tested, L1014F-VGSC being almost fixed everywhere, while bendiocarb resistance was limited to a few localities, G119S-*ace-1* remaining rare, with very limited variations during surveyed period. Interestingly, we found no effect of the type of insecticide pressure on the dynamics of these mutations.

**Conclusions:**

These results confirm both the high prevalence of PYR resistance and the potential of CXs/OPs as short- to medium-term alternatives in Benin. They also underline the need for regular resistance monitoring and informed management in their usage, as the G119S-*ace-1* mutation is already present in Benin and surrounding countries. Their unwise usage would rapidly lead to its spread, which would jeopardize PYR-resistant *Anopheles* control.
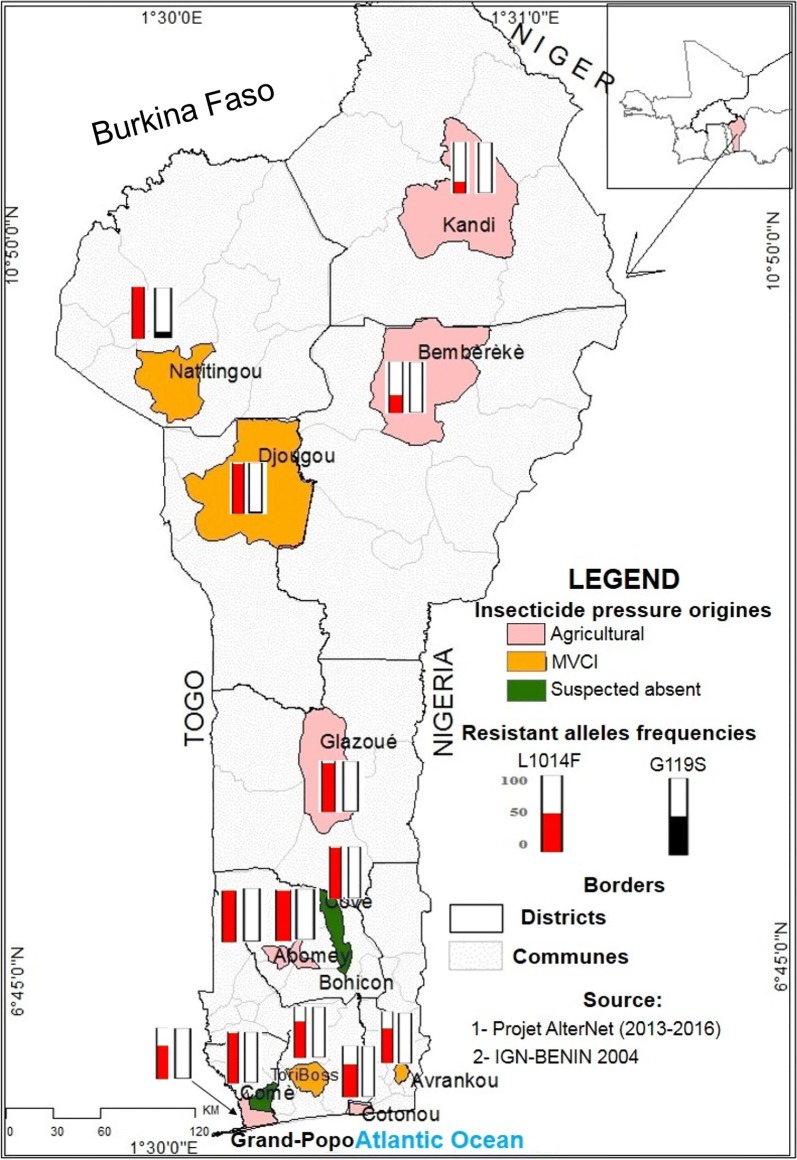

## Background

Controlling mosquito vectors of human diseases is a major health issue, particularly in sub-Saharan Africa where *Anopheles gambiae* (*s.l*.) is the main vector of malaria transmission. Malaria control is mainly based on insecticide use in the form of LLINs (long-lasting insecticide nets) and IRS (indoor residual spraying) [1]. Pyrethroid insecticides (PYRs) have been the cornerstone of malaria prevention in Africa for almost two decades, after Abuja Declaration in 2000, when Benin and the rest West African countries set-up a proper plan against malaria control [2]. However, the ability of vectors to survive insecticide treatments (i.e. become resistant) is a growing concern, as it may threaten vector control effectiveness for preventing malaria transmission [3–6]. Insecticide resistance is indeed widespread in natural populations in West Africa and more particularly in Benin, Burkina Faso and Ivory Coast [7–9].

In Benin, the Ministry of Health (MOH) and the National Malaria Control Programme (NMCP) started a large and extensive distribution programme of pyrethroid-treated LLINs across the country in 2008 [2]. Moreover, through the USA PMI programme (President Malaria Initiative), large-scale indoor spraying campaigns are implemented since 2008, using carbamate (CXs) and organophosphate (OPs) insecticides [10].

In addition to this insecticide pressure due to malaria vector control, insecticides are widely used in agriculture for crop protection. Agricultural pesticides often belong to the same classes as those used for vector control [7, 11]. Thus, they can affect the susceptibility of vector populations by increasing the frequencies of existing resistance mechanisms, or by selecting new mechanisms [11–14]. Because these selective pressures are heterogeneous, resistance frequency and its effect on vector control could vary from one population to another and over time [12].

This situation called the World Health Organization (WHO) to publish a Global Plan for Insecticide Resistance Management (GPIRM) [15] in 2012 (recently extended by guidelines at the national level), which emphasized the need for improved strategies for insecticide resistance management and prevention [16]. This objective cannot be achieved if the forces driving insecticide resistance evolution are not clearly understood and monitored.

Insecticide resistance dynamics, and thus long-term efficacy of vector control strategies, indeed depends on the biological characteristics of vectors, and the intensity and spatio-temporal distribution of selection pressures. It is therefore crucial to monitor the general patterns of resistance allele variations in relation with the various selective pressures that could drive these variations in vector populations. In the present study, we performed an updated overview of resistance status of *An. gambiae* (*s.l.*) in different agroclimatic zones with varying practices of insecticide across the whole Benin country. We acquired relevant information regarding selection pressure sources and its evolution to assess how they could contribute to the selection of two major target sites resistance mechanisms (the L1014F-VGSC and G119S-*ace-1* mutations, respectively for PYRs and CXs/OPs resistance). These two mutations are the major one involved into PYR and CXs/OPs resistant in *An. gambiae* (*s.l.*) in West Africa. However, other SNPs have been recently identified in the voltage gate sodium channel gene (VGSC), but data on their role in insecticide resistance are lacking [17, 18]. We provide encouraging data for CXs and OPs uses as means to circumvent the high *An. gambiae* (*s.l.*) PYR resistance in Benin for short- and medium-term.

## Methods

### Study sites and mosquito collections

**Fig. 1:**
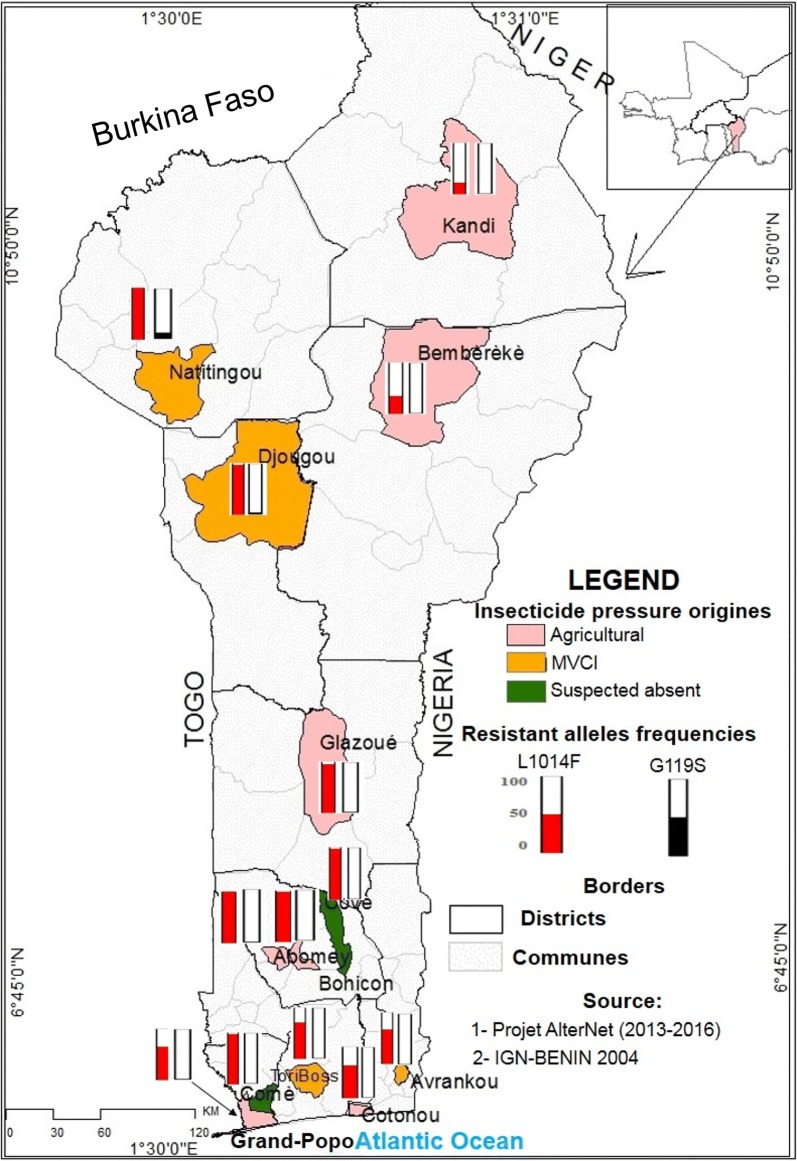
Map of the sample sites and insecticide resistant allele frequencies (L1014F-VGSC and G119S-*ace-1*) in natural populations of *An. gambiae* (*s.l.*) from of Benin. *Abbreviation*: MVCI, malaria vector control intervention

This study was carried out in 13 localities following a longitudinal transect across Benin (Fig. 1): five localities in the southern part, characterized by a Guinean bioclimate (two rainy seasons, April–July and September–November and an average annual rainfall of 1500 mm); four localities in the central part, characterized by a Sudano-Guinean bioclimate (two rainy seasons, April–July and September–November with an average annual rainfall of 1000 mm); and four localities in the northern part, characterized by a Sudanese bioclimate (one rainy season, June to October and an average annual rainfall of 900 mm). These localities were selected according to insecticide use for crops protection or for malaria vector control and priority was given to localities involved in previous studies, to assess resistance dynamics (Table 1): (i) seven localities (Cotonou, Grand-Popo, Bohicon, Abomey, Glazoué, Bembèrèkè and Kandi), where vegetables and cotton are produced, with strong economic interests for the farmers and government, are thus mainly exposed to agricultural treatments; (ii) four localities (Tori-Bossito, Avrankou, Djougou and Natitingou) are less exposed to these agricultural insecticides, but have been selected for specific experimental malaria vector control intervention (MVCI), with more intense vector control interventions, i.e. more frequent turnover of bednets and IRS implementation; and (iii) two localities have little insecticide exposure, with limited insecticides usage for domestic production or public-health (Covè and Comè).

**Table 1:** List of studied localities and their characteristics

Locality	Environment	Agricultural practice	Agroclimatic zone	Insecticide pressure origin	Reference
Cotonou	Urban	Vegetables	Guinean	Agriculture (OCs/OPs/PYRs)	[6]
Grand-Popo	Urban	Vegetables	Guinean	Agriculture (OCs/OPs/PYRs)	Present study
Bohicon	Urban	Cereals/Cotton	Soudano-Guinean	Agriculture (OCs/OPs/PYRs)	[6]
Abomey	Urban	Cereals/Cotton	Soudano-Guinean	Agriculture (OCs/OPs/PYRs)	[6]
Glazoue	Urban	Cereals	Soudano-Guinean	Agriculture (OCs/OPs/PYRs)	[6]
Kandi	Rural	Cotton	Soudanian	Agriculture (OCs/OPs/PYRs)	[6]
Bembèrèkè	Rural	Cotton	Soudanian	Agriculture (OCs/OPs/PYRs)	[6]
Tori-Bossito	Rural	Cereals/Fruits	Guinean	MVCI (CXs/PYRs)	[6]
Avrankou	Rural	Cereals	Guinean	MVCI (CXs/PYRs)	Present study
Djougou	Urban	Cotton	Soudanian	MVCI (CXs/PYRs)	[6]
Natitingou	Urban	Cotton	Soudanian	MVCI (OPs/CXs/PYRs)	[6]
Comé	Rural	Cereals	Guinean	Average	[11]
Covè	Rural	Cereals	Soudano-Guinean	Average	Present study

The collection period was from August to September in 2013, 2014 and 2015

*Notes:* OCs, PYRs, OPs and CXs correspond to organochlorines, pyrethroids, organophosphates and carbamates insecticides, respectively. MVCI corresponds to areas with reinforced malaria vector control interventions. Agriculture corresponds to areas where exposure to insecticides results mostly from their uses for crop cultures. Average corresponds to areas with limited insecticide uses for domestic agriculture and vector control (low turnover of impregnated bednets, see text)

*Anopheles gambiae* (*s.l.*) larvae were sampled in these localities over three years (2013–2015). Note that due to field conditions and availability of the larvae during the sampling campaign, we were not able to collect a sample in each locality each year. At least 2000 larvae were collected from different breeding sites within a 1-km radius, in order to have enough adults per locality for the different tests included into this study.

### Species identification

All emerged adult mosquitoes were assigned to the *An. gambiae* (*s.l.*) cryptic species complex using morphological and molecular tests [19–21] after DNA extraction from whole mosquito as described in Assogba et al. [22].

### Insecticide susceptibility bioassays of *An. gambiae* (*s.l.*) populations

Susceptibility assays were implemented according to the WHO protocol using 2- to 5-day-old mosquitoes [23]. When enough adults were available, four replicates of 20–25 mosquitoes per tube were exposed to filter papers impregnated with insecticide for 1 h supplied by the WHO Collaborating Centre, Universiti Sains Malaysia, Penang, Malaysia. After exposure, mosquitoes were transferred to a clean holding tube supplied with 10% sugar meal and mortality was recorded 24 h after exposure. Two insecticides were tested: permethrin (0.75%, a pyrethroid type I) and bendiocarb (0.1% carbamate). According to the WHO recommendations [23], the susceptibility status of each locality was accessed as susceptible, likely resistant and resistant, when observed mortality was 98–100%, 90–98% and less than 90%, respectively. After exposure to insecticide, survivors and dead mosquitoes were stored in silica gel for further analysis.

### Genotyping of L1014F-*VGSC* and G119S-*ace-1*

In *An. gambiae* (*s.l.*), the L1014F-VGSC and G119S-*ace-1* mutations are the major insecticide target-site resistance alleles for PYRs and CXs resistance, respectively. After exposure to permethrin (PYRs) or bendiocarb (CXs), survivors and dead individuals were genotyped using classical molecular tests [24, 25]. Moreover, 2263 individuals from the 13 localities, unexposed to insecticides, were also genotyped for these mutations to assess resistant alleles frequency dynamics in natural populations.

In populations where a heterozygote deficit was found, the *VGSC* locus was Sanger-sequenced to confirm VGSC genotypes using the Agd1 and Agd2 primers (293 bp PCR fragment), as described in [22]. We also sequenced (i) two resistant homozygotes *An. arabiensis* from Djougou (2015); (ii) one resistant homozygote *An. arabiensis*, one heterozygote and one resistant homozygote *An. coluzzii* from Bembèrèkè (2015); and (iii) one susceptible homozygote and one heterozygote *An. arabiensis*, as well as one susceptible homozygote *An. coluzzii* from Glazoué (2015).

### Statistical analyses

#### Data from bioassays

To test whether the mortality (Mortal) was impacted by several variables, a generalized linear mixed model (GLMM) was computed as: Mortal= Select + Year + Select:Year + (1| Pop) + ε, where Select is a 3-level factor indicating the nature of the insecticide pressure, Pop is a multi-level factor corresponding to the sampled population and Year is a 3-level factor corresponding to the year of sampling (Table 1); “:” indicates an interaction between factors, ε is the error parameter, following a binomial distribution. The Pop factor was computed as a random effect, because each population was exposed to only one kind of insecticide pressure. As bioassays were performed on adults and they were all morphological identified belonging to *An. gambiae* (*s.l.*), it was not possible to take into account the *Anopheles* species independently. The Pop factor thus includes inter-population differences in urbanness, bioclimate and species. The model was simplified as follows: significance of the different fixed terms was tested, starting from the interaction term, using likelihood ratio tests (LRT) corrected for overdispersion [26, 27] as described by Crawley [28]; non-significant terms (*P* > 0.05) were removed.

Fisherʼs exact test was used to compare the L1014F-*VGSC* and G119S-*ace-1* phenotypic distributions (i.e. number of RR, RS and SS individuals) between dead mosquitoes and survivors, independently in each population, each year and for each species. Sequential Bonferroni correction for multiple testing was used to identify potential false positives [29].

#### Population genetics analysis

To analyze the population genetic structure, we used Genepop version 4.2 online (http://genepop.curtin.edu.au). In the 2013–2015 samples, *F*_*IS*_ (assessing deviation from the panmictic equilibrium) were estimated independently in each species, each population and each year and *F*_*ST*_ (assessing differentiation between populations, i.e. population structure) independently for each year and species (according to Weir & Cockerham [30]).

To analyze the distribution and long-term dynamics of the resistance allele frequencies, L1014F-*VGSC* and G119S-*ace-1* genotyping data, collected in the same sampling sites between 2006 and 2010 and published in previous studies [7, 11, 31–35], or collected during this study (2013–2015), were combined. Multinomial log-linear models (*multinom* function in the R package *nnet*) were computed independently for each locus as: Geno  =  Pop + Year + Sp + Pop: Year + Pop: Sp + Year: Sp + Pop:Year:Sp + ε, where Geno is a 3-level response variable corresponding to numbers of individuals for each genotype RR, RS and SS, Pop is a multi-level factor corresponding to the sampled population, Year is a continuous variable corresponding to the year of sampling (to test for temporal variations) and Sp is a 3-level factor corresponding to the species (*arabiensis*, *gambiae* or *coluzzii*); “:” indicates an interaction between variables, ε is the error parameter, following a multinomial distribution.

These models were simplified as indicated above. When significant Year effect or Year:Pop interactions were detected, thus suggesting inter-annual variations of the genotype distribution, we analyzed each population of each species independently. We tested the significance of the Year variable by comparing (using LRT) the models Geno  =  Year + ε and Geno  =  1 + ε. Sequential Bonferroni correction for multiple testing was used to identify potential false positives [29]. All computations were performed using the R free software (v.3.3.3) [28].

## Results

### High permethrin resistance but low bendiocarb resistance

In Benin, exposure of *An. gambiae* (*s.l.*). populations to insecticides has two main origins: agriculture or vector control. During three years, we surveyed 13 populations from Benin, to assess their potential impacts on insecticide resistance dynamics (Table 1, Fig. 1). These localities were chosen because they presented strong contrasts in their main selective pressure. In crop production localities, agricultural usages of insecticides exceed by far the public-health exposure (“Agriculture” localities, Table 1). Other localities, with only limited crop farming, were chosen because vector control (“MVCI” localities, Table 1) is increased through more frequent distribution of impregnated bednets (1-year turnover) and indoor residual spraying in houses, so that public health is the main source of insecticides. Other localities were chosen because they present more limited insecticide usage (“Average” localities, Table 1). Note however, that agricultural usage for domestic production, as well as exposure to impregnated bednets (3-year turnover), are present in all these populations.

**Fig. 2:**
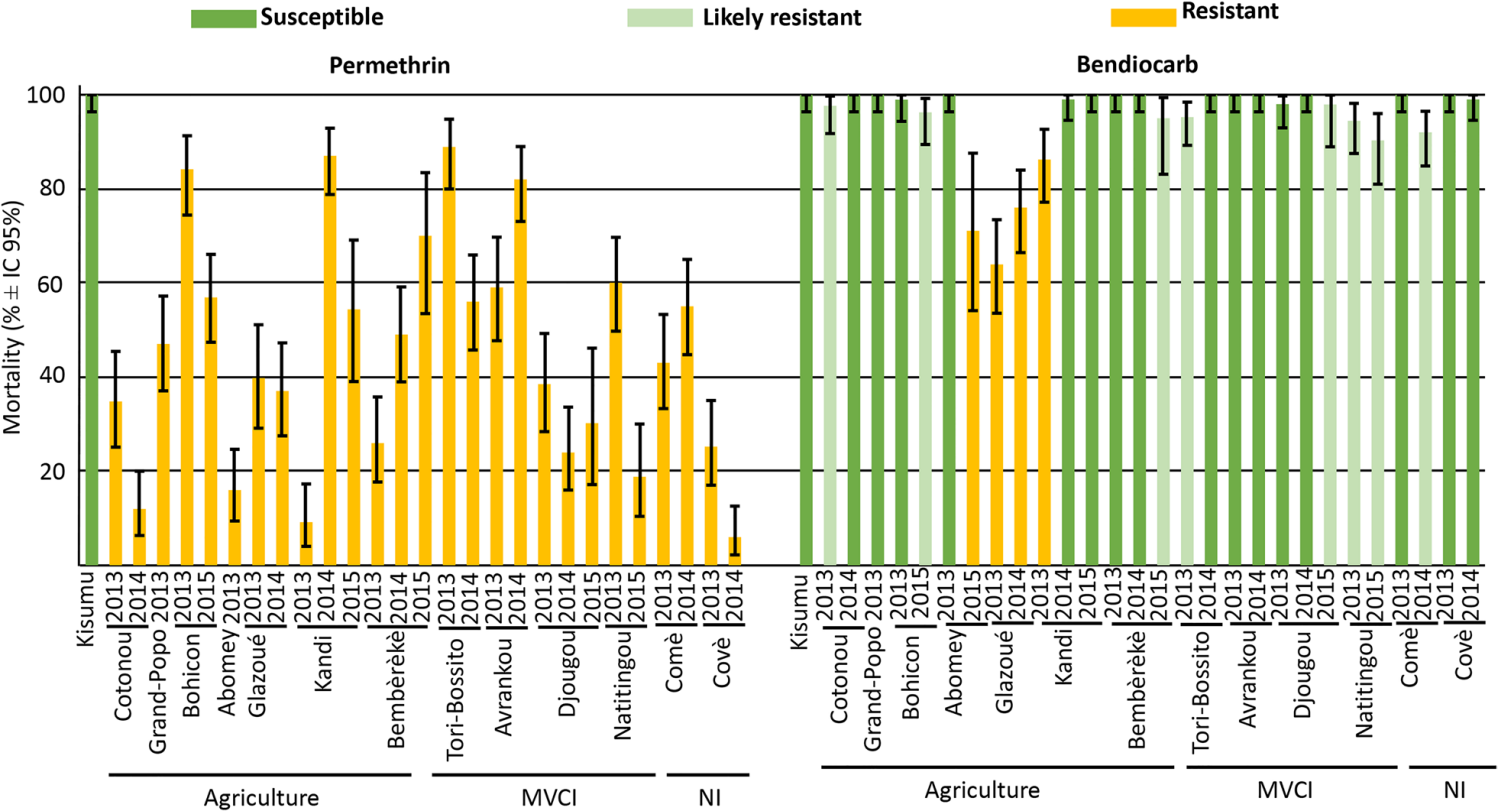
Mortality and susceptibility to insecticides status of *An. gambiae* (*s.l.*) from 13 localities of Benin sampled in September 2013, 2014 and 2015. The bars indicate the mortality percentage with 95% confidence intervals after one hour of exposure to impregnated paper in the WHO test kits and mortality reading after 24 h. The status “Susceptible”, “Likely resistant” and “Resistant” correspond to 98–100%, 90–98% and less than 90% mortality, respectively (WHO, 2016 [23]). Kisumu is the *An. gambiae* (*s.s.*) susceptible reference strain for the insecticides tested. The nature of the main insecticide exposure, agriculture, malaria vector control initiative (MVCI) or not identified (NI), is indicated

To first assess differences in resistance levels, larvae were collected in natural populations, emerged in the laboratory and 3–5-days-old females were exposed to permethrin or bendiocarb at the WHO discriminating doses. Mortality in control groups (i.e. unexposed to insecticides) was consistently < 5%, while the mortality of Kisumu individuals (the susceptible reference strain) was above 99% for both insecticides. All tested populations showed resistance to permethrin (mortality < 90%), but the mortality rate was highly variable between populations and between years (Fig. 2, permethrin).

We found a significant effect of the interaction between the nature of the main insecticide pressures and the collection year on mortality (GLMM, LRT corrected for overdispersion, *P* < 0.001; note that a large overdispersion, 3.01, was observed). Therefore, the effect of the nature of insecticide pressures on mortality was analyzed for each year independently. While no significant difference was found in 2013 and 2014 (GLMM, LRT, *P-value* > 0.05; no overdispersion), in 2015 the mortality in populations exposed to insecticides mostly from agriculture origins (Bohicon, Kandi and Bembèrèkè) was significantly higher than in populations exposed mostly to MVCI (Djougou and Natitingou) (GLMM, LRT, *P* < 0.001; no overdispersion).

Bendiocarb susceptibility was generally high (Fig. 2, bendiocarb), although some variability was observed between populations and between years. The interaction between the nature of the insecticide pressures and the collection year again showed a significant effect on mortality (GLMM, LRT corrected for overdispersion, *P*-value < 0.001; moderate overdispersion, 1.46). Analyzing each year independently, we found no significant effect of the nature of insecticide pressures on mortality (GLMM, LRT, *P*-value > 0.05; no overdispersion).

For each population independently, the dead or surviving larvae after exposure to permethrin or bendiocarb were genotyped for the L1014F-*VGSC* or G119S-*ace-1* mutations, respectively.

After exposure to permethrin, the frequency of L1014F-*VGSC* mutation was very high in both the dead and surviving individuals for all populations, usually over 0.7 (Additional file 1: Table S1). The L1014F-*VGSC* phenotypic distributions (i.e. numbers of RR, RS and SS individuals) were, however, significantly different between dead and surviving larvae overall (i.e. pooling all samples) for each year independently (Fisherʼs exact test on all dead and surviving larvae and all species, *P* < 0.001, Additional file 1: Table S3). However, there was no consistent difference between species or between years for any population (ex. for *Anopheles arabiensis*, we found no phenotypic distribution difference between dead and surviving larvae in 2013, significant differences in 2014, but again none in 2015; Additional file 1: Table S3).

After exposure to bendiocarb, there were overall very few individuals carrying the G119S-*ace-1* mutation in both the dead and surviving individuals for all populations and the mutation frequency was usually below 0.05 in dead and usually over 0.2 in surviving larvae (Additional file 1: Table S2). Again, there was no consistent G119S-*ace-1* phenotypic distribution difference between the dead and surviving larvae, be it between species or between years for any population (Additional file 1: Table S3).

### Heterogeneous resistance allele frequencies in natural populations of *An. gambiae* (*s.l.*)

A total of 2263 individuals collected from the same sites from 2013 to 2015, but unexposed to insecticides (50–60 from each site, Table 2) were then analyzed at the molecular level. We first assessed to which species these individuals belonged and found three *Anopheles* species, *An. coluzzii* (*n* = 1198), *An. gambiae* (*s.s.*) (*n* = 782) and *An. arabiensis* (*n* = 271), as well as very rare *coluzzii*-*gambiae* (*s.s.*) hybrids (*n* = 12) (see Table 2). These individuals were then screened for L1014F-*VGSC* and G119S-*ace-1* mutations to assess their frequencies and follow-up their evolution in natural populations.

**Table 2:** Frequencies of *L1014F-VGSC* and *G119S-ace-1* mutations over ten years (2006–2015) in *Anopheles gambiae* (*s.l.*) populations of Benin

Species	Locality	Year	Insecticide resistance alleles										Reference
			*G119S-ace-1*				*P-*value	*L1014F-VGSC*				*P-*value	
			SS	RS	RR	*f*		SS	RS	RR	*f*		
*An. arabiensis*	Bembèrèkè	2013	10	0	0	0	ns	7	0	3	0.30	ns	Present study
		2014	39	0	0	0		39	0	0	0		Present study
		2015	15	0	0	0		14	1	0	0.03		Present study
	Bohicon	2006	66	0	0	0	ns	66	0	0	0	ns	[6]
		2008	–	–	–	–		1	4	2	0.57		[29]
		2014	15	0	0	0		15	0	0	0		Present study
	Djougou	2014	1	0	0	0	ns	1	0	0	0	ns	Present study
		2015	3	0	0	0		0	0	3	1		Present study
	Glazoué	2007	57	0	0	0	ns	57	0	0	0	***	[6]
		2013	36	0	0	0		36	0	0	0		Present study
		2014	39	0	0	0		39	0	0	0		Present study
		2015	58	0	0	0		2	0	56	0.97		Present study
	Kandi	2007	32	0	0	0	ns	32	0	0	0	ns	[6]
		2013	16	0	0	0		13	0	3	0.19		Present study
		2014	38	0	0	0		36	0	2	0.05		Present study
		2015	1	0	0	0		1	0	0	0		Present study
*An. coluzzii*	Abomey	2006	3	0	0	0	ns	2	1	0	0.17	ns	[6]
		2013	6	0	0	0		0	0	6	1		Present study
		2014	50	3	0	0.03		0	0	53	1		Present study
		2015	29	0	0	0		0	0	29	1		Present study
	Avrankou	2013	35	2	0	0	ns	5	8	24	0.76	ns	Present study
		2014	59	1	0	0		3	13	44	0.84		Present study
		2015	60	0	0	0		10	20	30	0.67		Present study
	Bembèrèkè	2015	13	0	0	0	–	3	7	3	0.50	–	Present study
	Bohicon	2006	2	0	0	0	ns	0	0	2	1	***(ns)	[6]
		2008	–	–	–	–		5	1	1	0.21		[29]
		2010	–	–	–	–		1	0	11	0.92		[29]
		2013	44	0	0	0		1	3	40	0.94		Present study
		2014	28	0	0	0		1	3	24	0.91		Present study
		2015	25	0	0	0		0	1	24	0.98		Present study
	Comè	2009	30	0	0	0	ns	1	11	18	0.78	*(ns)	[30]
		2013	56	0	0	0		0	3	53	0.97		Present study
		2014	51	1	0	0.01		2	12	37	0.84		Present study
		2015	60	0	0	0		0	7	53	0.94		Present study
	Cotonou	2006	–	–	–	–	ns	93	140	67	0.46	***	[31]
		2007	92	0	0	0		3	13	79	0.90		[6]
		2008	–	–	–	–		3	5	31	0.86		[29]
		2009	72	0	0	0		3	29	97	0.86		[29, 30]
		2010	–	–	–	–		0	9	37	0.90		[29]
		2013	60	0	0	0		0	3	57	0.98		Present study
		2014	33	3	0	0.04		0	1	35	0.99		Present study
		2015	59	0	0	0		8	27	24	0.64		Present study
	Covè	2013	58	0	0	0	ns	0	11	47	0.91	ns	Present study
		2014	59	0	0	0		1	12	46	0.88		Present study
		2015	59	0	0	0		1	2	56	0.97		Present study
	Djougou	2015	1	0	0	0	–	1	0	0	0	–	Present study
	Glazoué	2013	1	0	0	0	ns	0	0	1	1	ns	Present study
		2014	4	0	0	0		1	0	3	0.75		Present study
		2015	1	0	0	0		1	0	0	0		Present study
	Grand-Popo	2013	56	7	0	0.06	ns	0	3	60	0.98	ns	Present study
		2014	60	0	0	0		1	21	38	0.81		Present study
		2015	57	1	0	0.01		11	19	28	0.65		Present study
	Kandi	2014	2	0	0	0	–	1	1	0	0.25	–	Present study
	Tori Bossito	2007	41	0	0	0	ns	33	8	0	0.10	***	[32]
		2008	62	0	0	0		33	16	13	0.34		[32]
		2009	–	–	–	–		0	5	25	0.92		[29]
		2010	–	–	–	–		0	14	16	0.77		[29]
		2013	60	0	0	0		0	2	58	0.98		Present study
		2014	36	0	0	0		1	6	29	0.89		Present study
		2015	58	0	0	0		5	24	29	0.71		Present study
*An. gambiae* (*s.s.*)	Abomey	2006	52	16	0	0.12	***	2	8	58	0.91	ns	[6]
		2013	47	2	0	0.02		0	0	49	1		Present study
		2014	4	0	0	0		0	0	4	1		Present study
		2015	30	1	0	0.02		0	0	31	1		Present study
	Avrankou	2013	22	1	0	0.02	–	3	5	15	1	–	Present study
	Bembèrèkè	2007	57	5	0	0.04	* (ns)	5	15	42	0.80	ns	[6]
		2013	47	0	0	0		0	1	46	0.99		Present study
		2014	21	0	0	0		4	0	17	0.81		Present study
		2015	19	0	0	0		6	8	5	0.47		Present study
	Bohicon	2006	2	1	0	0.17	ns	0	1	3	0.88	ns	[6]
		2008	–	–	–	–		3	6	16	0.76		[29]
		2009	–	–	–	–		0	4	41	0.96		[29]
		2010	–	–	–	–		0	7	26	0.89		[29]
		2013	15	0	0	0		0	0	15	1		Present study
		2014	14	0	0	0		0	1	13	0.96		Present study
		2015	35	0	0	0		0	2	33	0.97		Present study
	Comè	2013	4	0	0	0	–	0	0	4	1.00	–	Present study
	Covè	2014	1	0	0	0	ns	0	0	1	1.00	ns	Present study
		2015	1	0	0	0		0	0	1	1.00		Present study
	Djougou	2007	39	3	0	0.04	ns	1	10	31	0.86	ns	[6]
		2013	60	0	0	0		0	3	57	0.98		Present study
		2014	55	0	0	0		1	2	52	0.96		Present study
		2015	51	4	0	0.04		1	0	54	0.98		Present study
	Glazoué	2007	5	0	0	0	ns	4	1	0	0.10	ns	[6]
		2013	23	0	0	0		0	0	23	1		Present study
		2014	14	1	0	0.03		0	1	14	0.97		Present study
		2015	0	1	0	0.50		0	0	1	1		Present study
	Kandi	2007	30	0	0	0	ns	7	8	15	0.63	***	[6]
		2009	90	3	0	0.02		80	104	90	0.52		[11]
		2013	44	0	0	0		3	0	41	0.93		Present study
		2014	20	0	0	0		4	0	16	0.80		Present study
		2015	58	0	0	0		13	0	45	0.78		Present study
	Natitingou	2007	47	1	0	0.01	ns	1	13	47	0.88	***	[6]
		2012	–	–	–	–		33	11	0	0.13		[33]
		2013	60	0	0	0		0	0	60	1		Present study
		2014	46	3	0	0.03		0	0	49	1		Present study
		2015	46	12	0	0.10		0	0	58	1		Present study
	Tori Bossito	2007	144	2	0	0.01	ns	74	62	10	0.28	***	[32]
		2008	71	0	0	0		43	32	32	0.45		[29, 32]
		2009	–	–	–	–		0	5	20	0.90		[29]
		2010	–	–	–	–		1	5	5	0.68		[29]
		2014	20	0	0	0		0	0	20	1		Present study
*An. coluzii × An. gambiae* (*s.s.*) hybrids	Abomey	2013	5	0	0	0	–	0	0	5	1	–	Present study
	Bohicon	2013	1	0	0	0	–	0	0	1	1	–	Present study
	Bembèrèkè	2015	1	0	0	0	–	0	1	0	0.5	–	Present study
	Djougou	2015	1	0	0	0	–	0	0	1	1	–	Present study
	Tori Bossito	2014	4	0	0	0	–	0	0	4	1	–	Present study

*Notes:* For each species of the complex, the year of sampling and the number of individuals per genotype are indicated for both resistance mutations, as well as the source of the data. For each resistance mutation, the frequency of the resistant allele is calculated for each sample (*f*) and the P-value indicates whether there is a significant interannual variation (multinomial log-linear model, Year effect, see “Methods”): ns, *P* > 0.05, *P ≤ 0.05, **P ≤ 0.01, ***P ≤ 0.001 (when the Bonferroni correction suggests that the significant *P*-value is probably a false positive, ns is indicated within brackets). “–” is indicated when no data were available

**Fig. 3:**
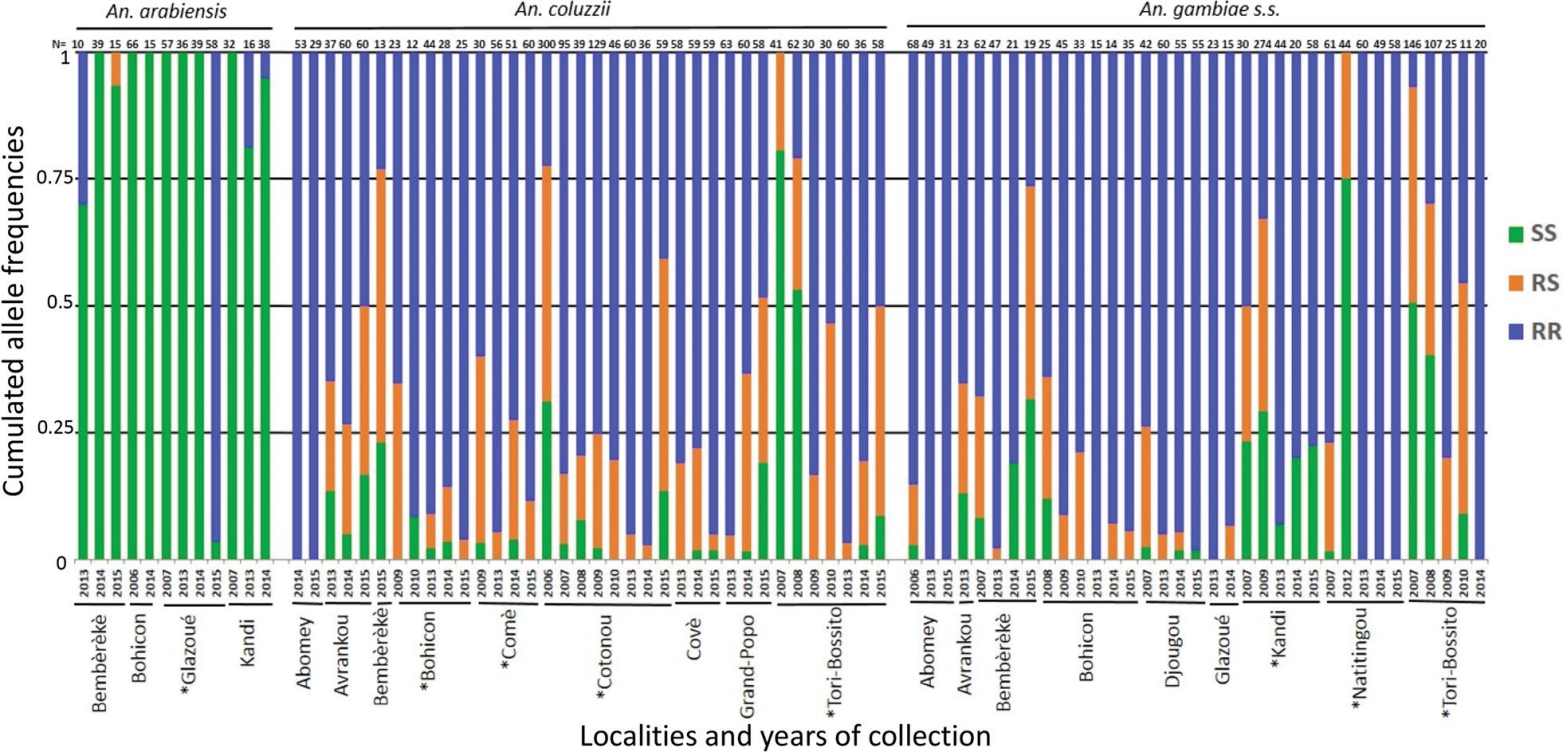
*L1014F-VGSC* genotype frequencies. The cumulated frequencies of SS, RS and RR genotypes are presented for each sample with more than 10 individuals analyzed (*N* ≥ 10). The locality and year of collection are also indicated (bottom), as well as the number of analyzed individuals (*N*) and the species (top). “*” indicates that the inter-annual variations are significant (multinomial log-linear model, Year effect, see “Methods”)

The L1014F-*VGSC* mutation was found in all three *Anopheles* species. However, its frequency was relatively low in *An. arabiensis* (0–0.3, with an average 0.21 ± 0.37, Fig, 3, Table 2), except in Glazoué in 2015, where the frequency was 0.97 (NB: genotype and species were confirmed by Sanger-sequencing three RR individuals). By contrast, the mutation was found in all localities and almost fixed in *An. coluzzii* and *An. gambiae* (*s.s.*), with average frequencies of 0.79 ± 0.27 and 0.93 ± 0.12, respectively (Fig. 3, Table 2). We then analyzed the population genetic structure (using *F* statistics in the sense of Weir & Cockerham [30]) and found that most populations were not different from a panmictic equilibrium (most *F*_*IS*_ were not statistically different from 0, Table 3), with significant differentiation between populations (*F*_*ST*_ < 0.2), that could reach high values as in 2015 (Table 3).

**Table 3:** Population genetics analyses at VGSC and *ace-1* locus

Species	Population	*ace-1*						*VGSC*					
		2013		2014		2015		2013		2014		2015	
		*F*_*IS*_		*F*_*IS*_		*F*_*IS*_		*F*_*IS*_		*F*_*IS*_		*F*_*IS*_	
*An. arabiensis*	Bèmbèrèkè	–		–		–		1	**	–		–	
	Bohicon	–		–		–		–		–		–	
	Djougou	–		–		–		–		–		–	
	Glazoué	–		–		–		–		–		1	***
	Kandi	–		–		–		1	***	1	***	–	
	*F*_*ST*_	*F*_*ST*_	–	*F*_*ST*_	–	*F*_*ST*_	–	*F*_*ST*_	0.211**	*F*_*ST*_	0.011 ns	*F*_*ST*_	0.917***
*An. coluzzii*	Abomey	–		− 0.02	ns	–		–		–		–	
	Avrankou	− 0.014	ns	–		–		0.432	*	0.195	ns	0.258	ns
	Bèmbèrèkè	–		–		–		–		–		− 0.037	ns
	Bohicon	–		–		–		0.38	ns	0.357	ns	–	
	Comè	–		–		–		− 0.019	ns	0.12	ns	− 0.054	ns
	Cotonou	–		− 0.03	ns	–		− 0.017	ns	–		0.021	ns
	Covè	–		–		–		− 0.097	ns	0.036	ns	0.489	ns
	Djougou	–		–		–		–		–		–	
	Glazoué	–		–		–		–		1	ns	–	
	Grand-Popo	− 0.051	ns	–		–		− 0.016	ns	− 0.121	ns	0.291	*
	Kandi	–		–		–		–		–		−	
	Tori-Bossito	–		–		–		− 0.009	ns	0.17	ns	0.010	ns
	*F*_*ST*_	*F*_*ST*_	0.033***	*F*_*ST*_	0.008 ns	*F*_*ST*_	–	*F*_*ST*_	0.078***	*F*_*ST*_	0.058***	*F*_*ST*_	0.165***
*An. gambiae* (*s.s.*)	Abomey	− 0.01	ns	–		–		–		–		–	
	Avrankou	–		–		–		0.421	ns	–		–	
	Bèmbèrèkè	–		–		–		–		1	***	0.182	ns
	Bohicon	–		–		–		–		–		− 0.015	ns
	Comè	–		–		–		–		–		–	
	Covè	–		–		–		–		–		–	
	Djougou	–		–		− 0.029	ns	− 0.017	ns	0.488	ns	1	**
	Glazoué	–		–		–		–		–		–	
	Kandi	–		–		–		1	ns	1	***	1	***
	Natitingou	–		− 0.021	ns	0.036	ns	–		–		–	
	Tori-Bossito	–		–		–		–		–		–	
	*F*_*ST*_	*F*_*ST*_	–	*F*_*ST*_	0.002 ns	*F*_*ST*_	0.082***	*F*_*ST*_	0.119***	*F*_*ST*_	0.082**	*F*_*ST*_	0.660***

*Notes*: *F*_*IS*_ is an estimation of deviation from the panmictic equilibrium in each species, each population and each year. *F*_*ST*_ is an estimation of differentiation between populations independently for each year and species (tested using Genepop option 3.3). ns, *P* > 0.05, *P ≤ 0.05, **P ≤ 0.01, ***P ≤ 0.001). “–” is indicated when no data are available

**Fig. 4:**
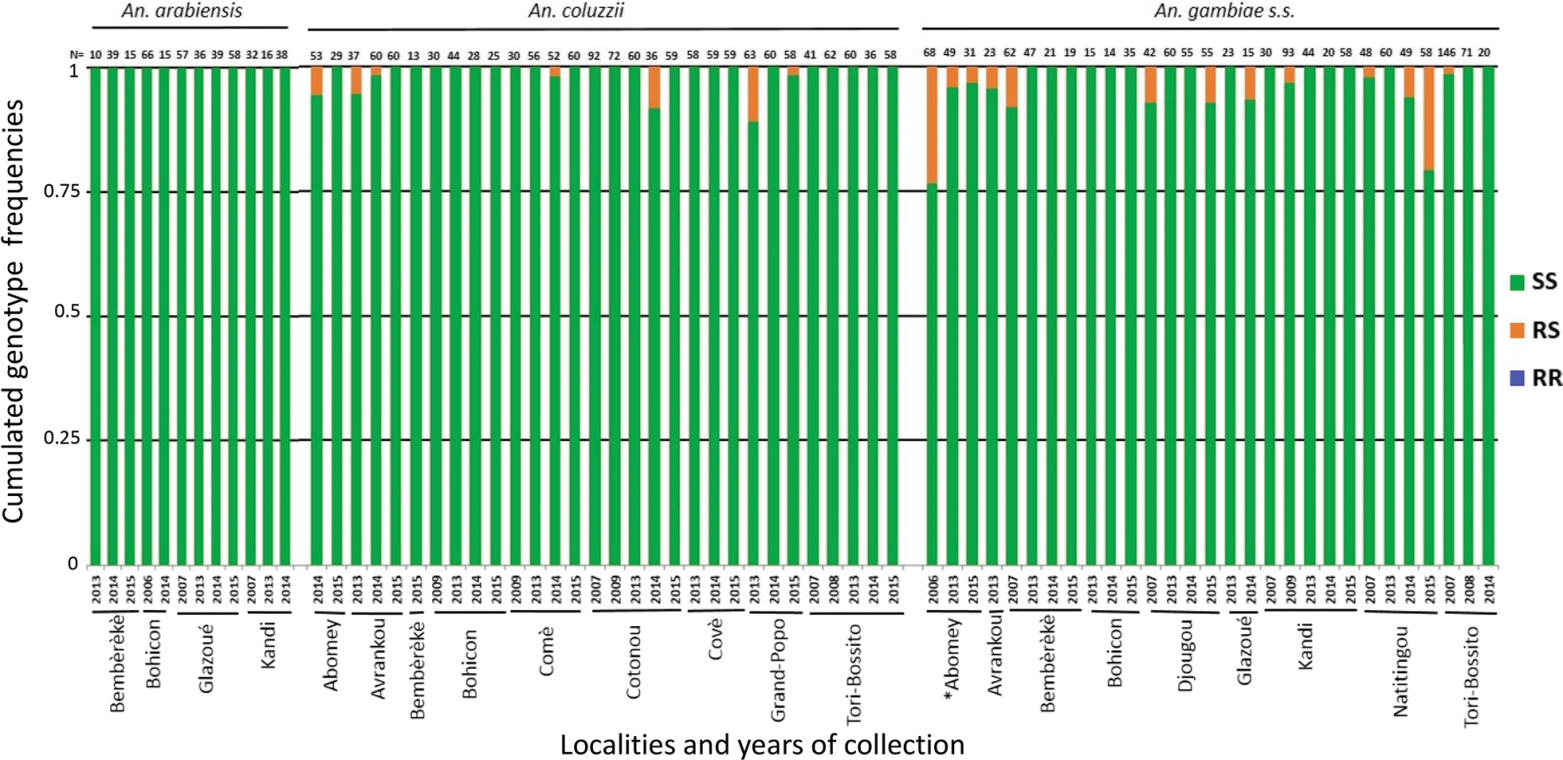
*G119S-ace-1* genotype frequencies. The cumulated frequencies of SS, RS and RR genotypes are presented for each sample with more than 10 individuals analyzed (*N* ≥ 10). The locality and year of collection are also indicated (bottom), as well as the number of analyzed individuals (*N*) and the species (top) “*” indicates that the inter-annual variations are significant (multinomial log-linear model, Year effect, see “Methods”)

The same samples were also screened for the G119S-*ace-1* mutation. A total of 271 *An. arabiensis* individuals carried the mutation; it was found in some populations for *An. coluzzii* and *An. gambiae* (*s.s.*), only in heterozygotes (Fig. 4). Frequencies were low (between 0–0.1, Table 2) and the mutation was often not detected in overall sampling years in some populations (Fig. 4). Due to the rarity of the mutation, most populations were SS monomorphic, which generally prevented the analysis of the *F*_*IS*_ (Tables 2, 3). Similarly, only low differentiation was found between populations (*F*_*ST*_ < 0.1, Table 3).

### Over ten years in Benin, *kdr* fluctuated at generally high frequencies, while *ace-1*^*R*^ remained rare

Adding data published from 2006 to 2010 in previous studies to the 2013–2015 new data, we analysed the distribution and long-term dynamics of the resistance allele frequencies, L1014F-*VGSC* and G119S-*ace-1.*

For L1014F-*VGSC*, multinomial log-linear models showed no significant Pop:Year:Sp interaction (LRT, *P*-value > 0.05), although all Pop:Year, Pop:Sp or Year:Sp interactions were significant (LRT, *P*-value < 0.001). We thus analysed the Year effect independently in each species and population (Table 2, Fig. 3). In *An. arabiensis*, only Glaouzé showed significant variations in resistance allele frequencies over the years, with a sharp increase in 2015 (*P*-value < 0.001, Table 2); in *An. coluzzii*, significant variations were found in Cotonou and Tori Bossito (with no clear trend, but slight and recent reductions after years at high frequencies) and tendencies to increase in Bohicon and Comè that did not, however, pass correction for multi-testing; in *An. gambiae* (*s.s.*), significant variations were found in Kandi (no clear trend, but a slight and recent reduction after years at high frequencies) and Naititingou and Tori Bossito (increases) (Table 2).

For G119S-*ace-1*, multinomial log-linear models showed no significant Pop:Year:Sp, Pop:Year, Pop:Sp or Year:Sp interactions (LRT, *P*-value > 0.05). Significant effects were, however, found for the simple factors Pop (LRT, *P*-value < 0.001), Sp (LRT, *P*-value < 0.01) and Year (LRT, *P*-value < 0.05). We again analyzed the Year effect independently in each species and population (Table 2, Fig. 4). Unsurprisingly considering the low mutation frequencies, we found no significant variations in resistance allele frequencies over the years, except in *An. gambiae* (*s.s.*) from Abomey, with no clear trend (Table 2).

## Discussion

Environmental variations induced by insecticides used against crop pests or vectors pathogens have been a strong selection pressure on vector populations. To understand how it affects these populations over time at both the phenotypic and genotypic levels, we investigated the susceptibility of *An. gambiae* (*s.l.*) to insecticides (permethrin and bendiocarb) and the distribution of the resistance target-site mutations (L1014F-VGSC and G119S-*ace-1*) in different agroclimatic zones, with different insecticide practices, across Benin. Indeed, climate and insecticide usage are key parameters in understanding the evolutionary dynamics of resistance genes.

### Pyrethroids resistance is frequent in all vector populations from Benin, driven by vector control and agriculture

One of the key questions of our study was to assess whether the origin of the insecticide pressures impacted the spread of resistance in vector populations from Benin.

We showed that all tested *An. gambiae* (*s.l.*) populations were consistently and often highly resistant to permethrin over the three consecutive years of our survey (Fig. 2, permethrin), which suggests that these mosquito populations are exposed to strong and pervasive pyrethroid insecticide pressures. Despite the difficulties of gathering information on the local treatment practices, we were able to compare populations exposed mainly to vector control treatments (MVCI) with populations exposed mainly to insecticides used for agriculture. As previous studies in Burkina Faso [2, 11], we found high PYR resistance in both kinds of localities, which strongly suggests that both practices are sources of selective pressure in Benin. There was, however, a high heterogeneity between populations, even under the same kind of treatment practices. This might be linked to the urban-ness or the bioclimate of the localities, although the number of populations investigated remains low and prevents definitive conclusions. We nevertheless found that, at least in 2015, MVCI resulted in significantly higher resistance levels than exposure to agriculture insecticides (Fig. 2).

In line with these observations at the phenotypic level, we also found high frequencies of the 1014F-*VGSC* mutation in the 2263 screened individuals from 13 localities over three years (Table 2, Fig. 3), confirming the tendency observed in previous studies [36] (see Table 2) or more recent years in southern Benin [37]. While the mutation frequency was high (and sometimes fixed) in both dead and survivor mosquitoes after 24 h exposure to permethrin (Additional file 1: Table S1), there were significant differences that tend to confirm the mutation adaptive advantage (Additional file 1: Table S3). Note however, that metabolic insecticide resistance mechanisms (e.g. P450-monooxigenase) have also been shown by previous studies to contribute to pyrethroid resistance in Benin [36, 38].

However, we show in this study that the distribution of the mutation in the different species of the *An. gambiae* (*s.l.*) complex is heterogeneous: while we found it in *An. coluzzii*, *An. gambiae* (*s.s.*), as well as in *An. arabiensis,* its frequency was much lower in the latter and higher in *An. gambiae* than in *An. coluzzii* (Fig. 3). This probably results from their ecological differences: while *An. arabiensis* is described as more zoophilic, exophagic and exophilic in savannah and sparse woodland, albeit with a high plasticity depending on the studies, including breeding in rice fields, *An. coluzzii* and *An. gambiae* (*s.s.*) are described as more anthropophilic, endophagic and endophilic (*An. gambiae* more so than *An. coluzzii*), again with variations [39, 40]. As such, *An. gambiae* (*s.s.*) and *An. coluzzii* (to a slightly lesser extent) would be more exposed than *An. arabiensis* to pyrethroids currently used in LLINs campaigns, which could explain the differences in L1014F-*VGSC* frequencies. Consequently, the higher prevalence of *An. arabiensis* in most populations exposed only to agriculture insecticides rather than MVCI (ex. Kandi, Glazoué, Bohicon and Bembèrèkè) could explain, at least in part, the higher mortality observed in bioassays (Figs. 2, 3).

Our study thus supports the notion that the type of insecticide exposure and the *Anopheles* species composition interact to explain the observed pyrethroid resistance levels in natural populations.

### L1014F-*VGSC* frequency tends to increase in Benin

To assess the dynamics of pyrethroid resistance, we combined data published in previous studies (2006–2010) [7, 11, 31–35] with the data collected for this study (2013–2015) and analyzed the long-term dynamics of the resistance allele frequency. The number of individuals analyzed was variable (Table 2), particularly when taking the species into account, so that the statistical power of our analyses was not optimal. Consequently, while there seems to be a general trend towards an increase in L1014F-*VGSC* frequency (Table 2, Fig. 3), we only found significant inter-annual variations for some of the populations, but in all three species (Fig. 3). This trend seems to persist in more recent samples (although the authors give regional rather than population-level data [37]). This persistence at high frequencies, despite its fitness costs, indicates that the resistance allele is still under selection (even if metabolic resistance mechanisms are also present [36, 38]). A similar situation has also been reported in *An. gambiae* (*s.l.*) populations from Burkina Faso, where over a ten-year period (between 1999 and 2009), the frequency of L1014F-*VGSC* mutation increased from 0.2 to 0.8 [41]. In this case, as in a study carried out on vegetable farming sites in southern of Benin [42], indirect evidence suggests that residues of PYRs/OCs in soils might select the L1014F-*VGSC* mutation, so that agricultural intensification would be one cause for these increases [41]. However, as shown above, vector control interventions are also responsible: a study in Mali indeed suggested that the increasing L1014F-*VGSC* frequency in *An. coluzzii* was due to the intensive use of long-lasting insecticidal nets [43]. Our results on the sharp increase in frequency L1014F-*VGSC* following the distribution of impregnated mosquito nets may reflect the same impact. It is important to note however, that significant decreases were also found in some populations (Table 2, Fig. 3): this shows that, despite the global trends, the local practices, both in agriculture and vector control, can rapidly impact the dynamics of the pyrethroid resistance allele. This heterogeneity in the practices also probably explains the high L1014F-*VGSC* frequency heterogeneities observed between populations (Additional file 1: Table S1). Overall, our study highlights the finesse of the data required to precisely follow, understand and predict resistance dynamics, but also encourages further investigations to acquire this knowledge as it shows that PYR resistance is not irreversible.

Moreover, this increase of PYR resistance mechanism may affect malaria parasite development within *An. gambiae* (*s.l.*) and thereby impact on malaria transmission. However, few studies have shown that the resistant vectors carrying L1014F-*VGSC* mutation increase the prevalence in oocysts and sporozoites development [44–46]. Moreover, studies designed to assess whether resistance has an impact on current vector control tools (LLINs and IRS), have shown the decrease of their effectiveness to kill resistant vectors [47–50]. Consequently, insecticide resistance could potentially maintain residual malaria transmission and jeopardize malaria elimination in many places of sub-Saharan Africa.

### Resistance to carbamates and organophosphates remains low in Benin, despite high prevalence in neighbouring countries

From 2013 to 2015, bendiocarb resistance of *An. gambiae* (*s.l.*) remained generally low in Benin, with very few localities showing less than 90% mortalities (Abomey, Glazoué and Kandi, Fig. 2, bendiocarb). Although all these localities are mostly exposed to agriculture insecticides rather than MVCI, the nature of insecticide pressures had no statistically significant effect. Note however, that the low resistance levels largely impeded the statistical power of the analyses.

Very few *An. arabiensis* surviving mosquitoes were obtained in bioassays, except in Glazoué and Cotonou and none carried the G119S mutation (Additional file 1: Table S2). This might signal a lack of adjustment for the bendiocarb diagnostic doses used in the WHO susceptibility tests for this species. It could also be due to the presence of metabolic resistance mechanisms like esterases, which have been described in *An. gambiae* from Burkina Faso, Ivory Coast, or Mayotte Island, Comoros [51–53]. There were only few survivors in bioassays for *An. coluzzii* and *An*. *gambiae* (*s.s.*) (Additional file 1: Table S2), with a higher, though still low, frequency of the G119S mutation (Fig. 4). Again, most surviving individuals did not carry this mutation, which could indicate the selection of other resistance mechanisms, such as detoxification.

**Fig. 5:**
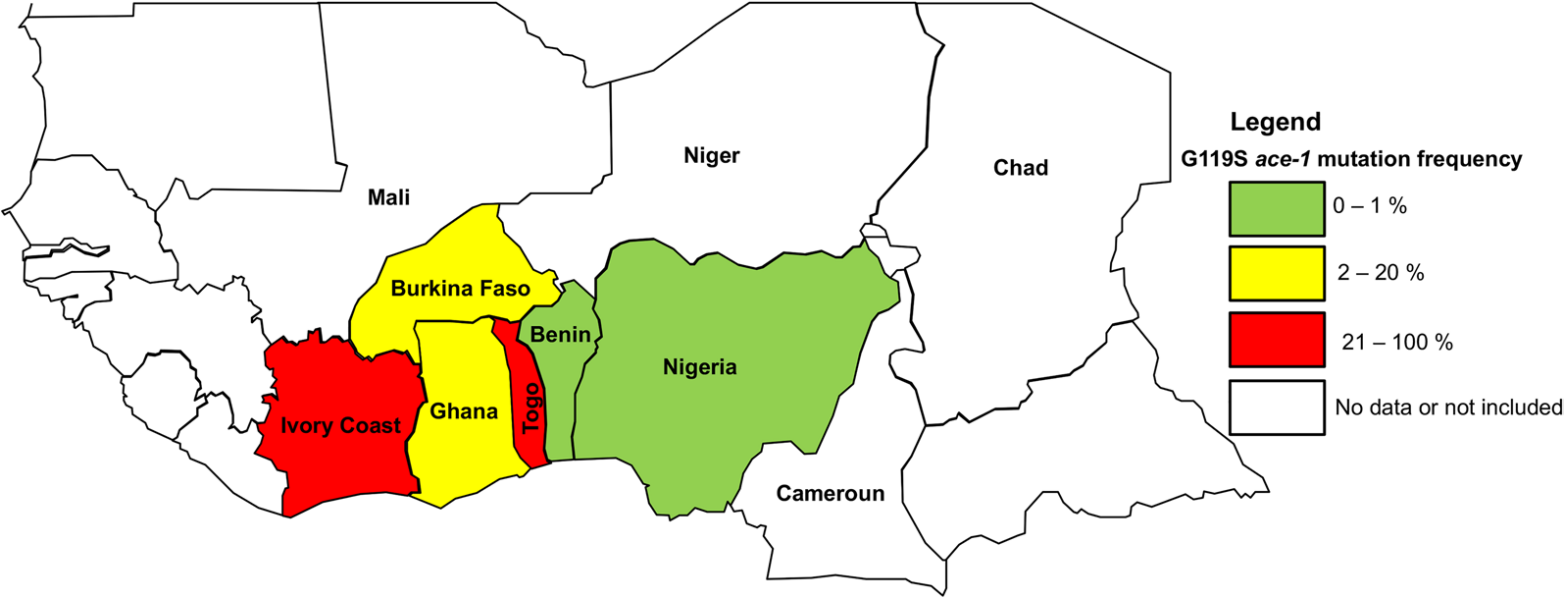
West-Africa map and G119S *ace-1* mutation frequency in natural populations of *Anopheles gambiae* (*s.l.*). Data were extracted from this study (Benin, 0.0095%), Assogba et al. (2018) [56] (Togo, 88.28%), Oduola et al. (2012) [57] (Nigeria, 0%), Essandoh et al. (2013) [58] (Ghana, 16.448%), Alou et al. (2010) [59] (Ivory Coast, 31.25%) and Dabiré et al. (2014) [60] (Burkina Faso, 18.54%)

When pooling data published in previous studies (2006–2010) with the new ones (2263 samples from 2013 to 2015), we confirmed that, although present in most, the mutation remains very rare in natural populations (Fig. 4) with no apparent geographical structure (Table 3). This is actually surprising as many surrounding countries display much higher G119S frequencies (Fig. 5), with no obvious reasons to explain the differences.

However, these results, high susceptibility and very low frequency of G119S-*ace-1* in vector populations, provide strong support for the proposed strategy of using CXs/OPs insecticides as an alternative to PYRs for vector control, at least in Benin. The pervasive and increasing PYR resistance in *An. gambiae* (*s.l.*) African populations indeed encouraged a switch from PYRs to CXs/OPs (or mixed applications) in several sub-Saharan African countries to preserve vector control effectiveness, with the support of the American President’s Malaria Initiative [10] and the National Malaria Control Programme (including Benin [54]). While this strategy appears interesting for short- or even medium terms, resistance will eventually spread, as the G119S-*ace-1* mutation is already present in vector populations and will be selected. A tight monitoring of the resistance markers is thus mandatory from the start, to prevent as long as possible and then try to limit the spread. Again, our study over ten years shows strong fluctuations, with detection of the mutation in some localities (Fig. 4, Table 2). This again suggests that a smart use of the insecticides could be implemented to moderate the selective pressure to slow the mutation spread and to withdraw insecticides in time to benefit from the selective cost associated with *G119S-ace-1* [22, 55]. It will in any case require fine monitoring and anticipation from the different actors involved in vector control, scientists and programme managers, to prevent repeating the previous decisions that led to the PYR resistance situation.

## Conclusions

Our results confirm both the high prevalence of PYR resistance and the potential of CXs/OPs as short- to medium term alternatives in Benin. They also underline the need for regular resistance monitoring and informed management in their usage, as the G119S-*ace-1* mutation is already present in Benin and surrounding countries. Unwise usage of CXs/OPs would rapidly lead to its spread, which would jeopardize PYR-resistant *Anopheles* control.

## Supplementary information

**Additional file 1: Table S1.** L1014F-VGSC allele frequency in survivor and dead *Anopheles gambiae* (*s.l*.) after 1-hour exposure to permethrin insecticide. **Table S2.** G119S-ace-1 allele frequency in survivor and dead of *Anopheles gambiae* (*s.l.*) after 1-hour exposure to bendiocarb insecticide. **Table S3.***P*-values of Fisherʼs exact tests comparing the phenotypic distributions (i.e. numbers of SS, RS and RR individuals) between dead and surviving mosquitoes for the L1014F-VGSC and G119S-ace-1 mutations, after exposure respectively to permethrin and bendiocarb insecticides. *P*-values < 0.05 are bolded when still significant after sequential Bonferroni correction, italicized otherwise. NB: only populations where more than one phenotype was found are considered here.

## Data Availability

All data generated or analysed during this study are included in this published article and its additional file.

## Abbreviations

*s.l.*: *sensu lato*
*s.s.*: *sensu stricto*
RR: homozygote-resistant
RS: heterozygote
SS: homozygote susceptible
PYRs: pyrethroids
CXs: carbamates
OPs: organophosphates
L1014F-VGSC: mutation leucine by phenylalanine at 1014 position in voltage-gated sodium channel protein
G119S-*ace-1*: mutation glycine by serine at position 119 in acetylcholinesterase 1 enzyme
GLMM: generalized linear mixed model
LRT: likelihood ratio tests
*F*_*IS*_: proportion of the variance in the subpopulation contained in an individual
*F*_*ST*_: proportion of the total genetic variance contained in a subpopulation (the S subscript) relative to the total genetic variance (the T subscript)
MOH: Ministry of Health
NMCP: National Malaria Control Programme
GPIRM: Global Plan for Insecticide Resistance Management
MVCI: Malaria Vector Control Intervention
WHO: World Health Organization
