# Supplementary material for: Dynamic of resistance alleles of two major insecticide targets in Anopheles gambiae (s.l.) populations from Benin, West Africa
Source: Parasit Vectors. 2020 Mar 14;13:134. doi: 10.1186/s13071-020-4006-6 (PMC7071764; doi:10.1186/s13071-020-4006-6)
Supplement: Supplementary file 1 — Additional file 1: Table S1. L1014F-VGSC allele frequency in survivor and dead Anopheles gambiae (s.l.) after 1-hour exposure to permethrin insecticide. Table S2. G119S-ace-1 allele frequency in survivor and dead of Anopheles gambiae (s.l.) after 1-hour exposure to bendiocarb insecticide. Table S3.P-values of Fisherʼs exact tests comparing the phenotypic distributions (i.e. numbers of SS, RS and RR individuals) between dead and surviving mosquitoes for the L1014F-VGSC and G119S-ace-1 mutations, after exposure respectively to permethrin and bendiocarb insecticides. P-values < 0.05 are bolded when still significant after sequential Bonferroni correction, italicized otherwise. NB: only populations where more than one phenotype was found are considered here. [file 13071_2020_4006_MOESM1_ESM.pdf]

Dynamic of resistance alleles of two major insecticide targets in *Anopheles gambiae s.l.* populations from Benin, West Africa

Benoît S. Assogba[1,2,3,4*], Nicole Pasteur[1], Patrick Makoundou[1], Sandra Unal[1], Lamine Baba-Moussa[3], Pierrick Labbé[1] and Mylène Weill[1]

Additional file 1

Table S1: *L1014F-VGSC* allele frequency in survivor and dead *Anopheles gambiae (s.l.)* after 1-hour exposure to permethrin insecticide

Localities	Year	Species	Dead					Survivor				
			N	SS	RS	RR	*f*	N	SS	RS	RR	*f*
Cotonou	2013	*An. coluzzii*	25	3	5	17	0.78	25	0	3	22	0.94
Grand-Popo	2013	*An. coluzzii*	25	3	2	20	0.84	25	1	0	24	0.96
Bohicon	2013	*An. coluzzii*	16	0	1	15	0.96	4	0	1	3	0.87
		An. gambiae s.s.	9	0	0	9	1	9	0	1	8	0.94
Abomey	2013	*An. coluzzii*	2	0	0	2	1	4	0	0	4	1
		An. gambiae s.s.	14	0	1	13	0.96	21	0	0	21	1
Glazoué	2013	*An. arabiensis*	11	9	0	2	0.18	8	8	0	0	0
		An. gambiae s.s.	14	0	1	13	0.96	17	1	0	16	0.94
Kandi	2013	*An. arabiensis*	8	8	0	0	0	12	11	0	1	0.08
		An. gambiae s.s.	-	-	-	-	-	13	1	0	12	0.92
Bembèrèkè	2013	*An. arabiensis*	-	-	-	-	-	1	1	0	0	0
		An. gambiae s.s.	25	4	0	21	0.84	24	1	0	23	0.95
Tori-Bossito	2013	*An. coluzzii*	20	1	2	17	0.9	9	0	2	7	0.88
		An. gambiae s.s.	5	0	0	5	1	-	-	-	-	-
Avrankou	2013	*An. coluzzii*	14	1	5	8	0.75	16	1	3	12	0.84
		An. gambiae s.s.	11	0	4	7	0.81	9	0	1	8	0.94
Djougou	2013	*An. gambiae s.s.*	25	0	0	25	1	25	0	0	25	1
Natitingou	2013	*An. gambiae s.s.*	25	0	0	25	1	25	0	0	25	1
Comè	2013	*An. coluzzii*	19	0	2	17	0.94	19	0	0	19	1
		An. gambiae s.s.	6	0	1	5	0.91	6	0	0	6	1
Covè	2013	*An. coluzzii*	22	0	9	13	0.79	25	1	1	23	0.94
		An. gambiae s.s.	3	0	1	2	0.83	-	-	-	-	-
Cotonou	2014	*An. coluzzii*	12	0	1	11	0.95	25	0	4	21	0.92
Glazoué	2014	*An. arabiensis*	23	23	0	0	0	20	19	0	1	0.05
		An. gambiae s.s.	2	1	0	1	0.5	5	0	0	5	1
Kandi	2014	*An. arabiensis*	17	4	0	13	0.76	5	5	0	0	0
		An. gambiae s.s.	8	0	0	8	1	8	1	0	7	0.87
Bembèrèkè	2014	*An. arabiensis*	11	9	0	2	0.18	9	9	0	0	0
		An. gambiae s.s.	14	0	0	14	1	16	0	0	16	1
Tori-Bossito	2014	*An. coluzzii*	16	1	3	12	0.84	18	0	0	18	1
		An. gambiae s.s.	9	0	0	9	1	7	0	0	7	1
Avrankou	2014	*An. coluzzii*	25	1	17	7	0.62	18	0	0	18	1
Djougou	2014	*An. gambiae s.s.*	24	0	0	24	1	25	0	0	25	1
Comè	2014	*An. coluzzii*	25	1	1	23	0.94	25	0	1	24	0.98
Covè	2014	*An. coluzzii*	6	0	2	4	0.83	25	0	5	20	0.9
Bohicon	2015	*An. coluzzii*	20	0	0	20	1	39	0	0	39	1
	2015	*An. gambiae s.s.*	28	0	0	28	1	10	0	0	10	1
Djougou	2015	*An. gambiae s.s.*	13	0	0	13	1	30	0	0	30	1
Natitingou	2015	*An. gambiae s.s.*	12	0	1	11	0.95	54	0	0	54	1
Bembèrèkè	2015	*An. gambiae s.s.*	12	10	0	2	0.16	6	4	1	1	0.25
	2015	*An. arabiensis*	13	2	2	9	0.76	6	0	1	5	0.91
Kandi	2015	*An. gambiae s.s.*	-	-	-	-		1	1	0	0	0
	2015	*An. arabiensis*	25	1	5	19	0.86	20	0	2	18	0.95

Notes: N is number of dead or surviving individuals genotyped for *L1014F-VGSC* mutation. RR, RS and SS correspond respectively to homozygous resistant, heterozygous and homozygous susceptible genotypes. *f* is the frequency of the mutation.

Table S2: *G119S-ace-1* allele frequency in survivor and dead of *Anopheles gambiae (s.l.)* after 1-hour exposure to bendiocarb insecticide

Localities	Year	Species	Dead					Survivor				
			N	SS	RS	RR	*f*	N	SS	RS	RR	*f*
Cotonou	2013	*An. coluzzii*	40	40	0	0	0	2	2	0	0	0
Grand-Popo	2013	*An. coluzzii*	40	40	0	0	0	-	-	-	-	-
Bohicon	2013	*An. coluzzii*	29	29	0	0	0	1	1	0	0	0
		An. gambiae s.s.	11	11	0	0	0	-	-	-	-	-
Abomey	2013	*An. coluzzii*	10	10	0	0	0	-	-	-	-	-
		An. gambiae s.s.	30	30	0	0	0	-	-	-	-	-
Glazoué	2013	*An. coluzzii*	2	2	0	0	0	-	-	-	-	-
		An. arabiensis	24	24	0	0	0	35	35	0	0	0
		An. gambiae s.s.	14	13	1	0	0.04	-	-	-	-	-
Kandi	2013	*An. arabiensis*	13	13	0	0	0	3	3	0	0	0
		An. gambiae s.s.	27	27	0	0	0	9	9	0	0	0
Bembèrèkè	2013	*An. arabiensis*	7	7	0	0	0	-	-	-	-	-
		An. gambiae s.s.	33	33	0	0	0	-	-	-	-	-
Tori-Bossito	2013	*An. coluzzii*	40	39	1	0	0.01	5	5	0	0	0
Avrankou	2013	*An. coluzzii*	32	31	0	0	0	-	-	-	-	-
		An. gambiae s.s.	8	9	0	0	0	-	-	-	-	-
Djougou	2013	*An. gambiae s.s.*	40	40	0	0	0	2	2	0	0	0
Natitingou	2013	*An. gambiae s.s.*	40	40	0	0	0	6	0	5	1	0.58
Comè	2013	*An. coluzzii*	25	25	0	0	0	-	-	-	-	-
		An. gambiae s.s.	15	15	0	0	0	-	-	-	-	-
Covè	2013	*An. coluzzii*	40	40	0	0	0	-	-	-	-	-
Cotonou	2014	*An. coluzzii*	40	40	0	0	0	-	-	-	-	-
Glazoué	2014	*An. arabiensis*	33	33	0	0	0	22	22	0	0	0
		An. coluzzii	1	1	0	0	0	-	-	-	-	
		An. gambiae s.s.	7	7	0	0	0	1	0	1	0	0.5
Kandi	2014	*An. arabiensis*	26	26	0	0	0	1	1	0	0	0
		An. coluzzii	2	2	0	0	0	-	-	-	-	-
		An. gambiae s.s.	12	11	1	0	0.04	-	-	-	-	-
Bembèrèkè	2014	*An. arabiensis*	6	6	0	0	0	-	-	-	-	-
		An. gambiae s.s.	32	30	2	0	0.03	-	-	-	-	-
Tori-Bossito	2014	*An. coluzzii*	21	20	1	0	0.02	-	-	-	-	-
		An. gambiae s.s.	19	19	0	0	0	-	-	-	-	-
Avrankou	2014	*An. coluzzii*	40	40	0	0	0	-	-	-	-	-
Djougou	2014	*An. arabiensis*	1	1	0	0	0	-	-	-	-	-
		An. gambiae s.s.	39	39	0	0	0	-	-	-	-	-
Comè	2014	*An. coluzzii*	34	34	0	0	0	8	8	0	0	0
		An. gambiae s.s.	5	5	0	0	0	-	-	-	-	-
Covè	2014	*An. coluzzii*	39	38	1	0	0.01	1	1	0	0	0
		An. gambiae s.s.	-	-	-	-	-	1	1	0	0	0

Localities	Year	Species	Dead					Survivor				
			N	SS	RS	RR	*f*	N	SS	RS	RR	*f*
Djougou	2015	*An. coluzzii*	1	1	0	0	0	-	-	-	-	-
		An. gambiae s.s.	41	41	0	0	0	1	0	1	0	0.5
Natitingou	2015	*An. gambiae s.s.*	45	45	0	0	0	7	3	4	0	0.28
Abomey	2015	*An. coluzzii*	1	1	0	0	0	1	1	0	0	0
		Hybrid	-	-	-	-	-	1	1	0	0	0
		An. gambiae s.s.	23	23	0	0	0	9	0	7	2	0.61
Bembèrèkè	2015	*An. arabiensis*	17	17	0	0	0	-	-	-	-	-
		An. coluzzii	7	7	0	0	0	2	0	2	0	0.5
		Hybrid	2	2	0	0	0	-	-	-	-	-
		An. gambiae s.s.	7	7	0	0	0	-	-	-	-	-
Bohicon	2015	*An. coluzzii*	15	15	0	0	0	1	1	0	0	0
		An. gambiae s.s.	33	33	0	0	0	2	1	1	0	0.25
Kandi	2015	*An. gambiae s.s.*	1	1	0	0	0	-	-	-	-	-
		An. arabiensis	41	41	0	0	0	-	-	-	-	-

Notes: N is number of dead or surviving individuals genotyped for *G119S-ace-1* mutation. RR, RS and SS correspond respectively to homozygous resistant, heterozygous and homozygous susceptible genotypes. *f* is the frequency of the mutation.

Table S3: *P*-values of Fisher's Exact tests comparing the phenotypic distributions (*i.e.* numbers of SS, RS and RR individuals) between dead and survivor mosquitoes for the L1014F-*VGSC* and G119S-*ace-1* mutations, after exposure respectively to permethrin and bendiocarb insecticides. *P*-values <0.05 are bolded when still significant after sequential Bonferroni correction, italicized otherwise. NB: only populations where more than one phenotype was found are considered here.

Year	Species	Populations	*P*-value	
			VGSC	*ace-1*
2013	All		**5.10^{-4}**	**1.10^{-6}**
	An. arabiensis	all	0.438	**3.10^{-4}**
		Glazoué	0.485	-
		Kandi	1	-
	An. coluzzi	all	**0.006**	0.109
		Avrankou	0.696	-
		Bohicon	0.368	-
		Comè	0.486	-
		Cotonou	0.141	-
		Covè	*0.003*	-
		Grand-Popo	0.221	-
		Tori-Bossito	0.706	1
	An. gambiae	all	**0.008**	**1.10^{-5}**
		Abomey	0.4	-
		Avrankou	0.319	-
		Bembèrèkè	0.349	-
		Bohicon	1	-
		Comè	1	-
		Covè	1	-
		Glazoué	0.707	1
		Kandi	1	-
		Natitingou	-	**1.10^{-7}**
2014	All		**1.10^{-6}**	**1.10^{-6}**
	An. arabiensis	all	**0.004**	**5.10^{-4}**
		Bembèrèkè	0.479	-
		Glazoué	0.465	-
		Kandi	*0.005*	-
	An. coluzzi	all	**1.10^{-5}**	**0.006**
		Avrankou	**1.10^{-6}**	-
		Comè	1	-
		Cotonou	1	-
		Covè	0.596	1

		Tori-Bossito	*0.04*	1
	An. gambiae	all	0.597	**0.007**
		Bembèrèkè		1
		Glazoué	0.286	0.25
		Kandi	1	1
2015	All		**1.10^{-6}**	**1.10^{-6}**
	An. arabiensis	all	0.736	1
		Bembèrèkè	1	-
		Kandi	0.54	-
	An. coluzzi	all	-	**0.01**
		Bembèrèkè	-	*0.028*
	An. gambiae	all	**1.10^{-5}**	**1.10^{-5}**
		Abomey	-	**4.10^{-8}**
		Bohicon	-	0.057
		Bembèrèkè	0.676	-
		Djougou	-	*0.024*
		Natitingou	0.182	**1.10^{-4}**
